# A systematic review of cluster randomised trials in residential facilities for older people suggests how to improve quality

**DOI:** 10.1186/1471-2288-13-127

**Published:** 2013-10-22

**Authors:** Karla Diaz-Ordaz, Robert Froud, Bart Sheehan, Sandra Eldridge

**Affiliations:** 1 Centre for Primary Care and Public Health, Queen Mary University of London, London, E1 2AB, UK; 2 Department of Health Services Research and Policy, London School of Hygiene and Tropical Medicine, 15 - 17 Tavistock Place,London, WC1H 9SH, UK; 3 University College of Health Sciences, Campus Kristiania, Oslo, Norway; 4 John Radcliffe Hospital, Oxford, OX3 9DU, UK

**Keywords:** Residential facilities, Older people, Cluster randomised trials

## Abstract

**Background:**

Previous reviews of cluster randomised trials have been critical of the quality of the trials reviewed, but none has explored determinants of the quality of these trials in a specific field over an extended period of time. Recent work suggests that correct conduct and reporting of these trials may require more than published guidelines. In this review, our aim was to assess the quality of cluster randomised trials conducted in residential facilities for older people, and to determine whether (1) statistician involvement in the trial and (2) strength of journal endorsement of the Consolidated Standards of Reporting Trials (CONSORT) statement influence quality.

**Methods:**

We systematically identified trials randomising residential facilities for older people, or parts thereof, without language restrictions, up to the end of 2010, using National Library of Medicine (Medline) via PubMed and hand-searching. We based quality assessment criteria largely on the extended CONSORT statement for cluster randomised trials. We assessed statistician involvement based on statistician co-authorship, and strength of journal endorsement of the CONSORT statement from journal websites.

**Results:**

73 trials met our inclusion criteria. Of these, 20 (27%) reported accounting for clustering in sample size calculations and 54 (74%) in the analyses. In 29 trials (40%), methods used to identify/recruit participants were judged by us to have potentially caused bias or reporting was unclear to reach a conclusion. Some elements of quality improved over time but this appeared not to be related to the publication of the extended CONSORT statement for these trials. Trials with statistician/epidemiologist co-authors were more likely to account for clustering in sample size calculations (unadjusted odds ratio 5.4, 95% confidence interval 1.1 to 26.0) and analyses (unadjusted OR 3.2, 1.2 to 8.5). Journal endorsement of the CONSORT statement was not associated with trial quality.

**Conclusions:**

Despite international attempts to improve methods in cluster randomised trials, important quality limitations remain amongst these trials in residential facilities. Statistician involvement on trial teams may be more effective in promoting quality than further journal endorsement of the CONSORT statement. Funding bodies and journals should promote statistician involvement and co-authorship in addition to adherence to CONSORT guidelines.

## Background

In cluster randomised trials, groups (or clusters) of individuals are randomised, rather than the individuals themselves. Over the past 25 years this trial design has become increasingly common in many fields such as primary care, where the clusters are often general practices (because the target of an intervention is a practice or its staff), and in low-income countries (where villages or geographic areas are often randomised to avoid contamination between intervention and control group participants in the same village/area). These trials are more complicated than individually randomised trials to design, conduct and analyse. In particular, sample size calculations and analyses should be adjusted to allow for homogeneity of individuals in the clusters, and investigators must avoid potential bias when identifying and recruiting individual participants [1-4]. Previous reviews of these trials indicate low quality in respect of these and other methodological and reporting criteria [4-12], and a possible trend from comparing review results of quality improvement over time [4]. Only one previous review explores trends in and determinants of quality directly [12], finding generally higher quality in journals with higher impact factors, but only modest impact of the 2004 extended CONSORT statement for cluster randomised trials [13]. In that paper, the authors suggest that it may require more than publication of guidelines to assist editors and investigators in the correct conduct and reporting of these trials. Elsewhere it has been hypothesised that pressure from statisticians may have contributed to improvements in the quality of these trials [9]. Whilst there is no empirical evidence to support this, there is evidence for the effect of the presence of statisticians on the quality of individually randomised trials [14,15]. Previous research has identified better reporting amongst randomised controlled trials published in journals more strongly promoting the 1996 original CONSORT statement [16].

We were aware of several cluster randomised trials evaluating a range of interventions in residential facilities for older people [17,18]. We reasoned that these trials should be common in such facilities because of the types of intervention likely to be evaluated such as changing treatment policies, and because of the risk of contamination between individuals living in such close proximity even when an individually randomised trial might have been possible in other settings. There are no previous reviews of trials in this area; we therefore expected quality to be more variable than in areas where the publication of reviews may have influenced conduct and reporting. There are ageing populations worldwide and even the most optimistic projections for community focused care suggest that the proportion of over 65s that can expect to receive long-term care in residential facilities will increase [19,20] beyond current levels. About a half are currently in long-term care [21,22]. The quality of cluster randomised trials in care homes is therefore important for shaping future healthcare evidence [23].

Here we describe the characteristics, and quality of methods and of reporting, of cluster randomised trials in residential facilities and provide some practical guidance for future investigators that could facilitate faster improvement in trial design, conduct and analysis, and thus accelerate an increase in the quantity of high quality research in this area. In particular, we wanted to see whether statistician involvement in the trial, and the strength of a journal’s endorsement of the CONSORT statement affected the methodological and reporting quality.

## Methods

### Inclusion and exclusion criteria

We included cluster randomised trials conducted up to the end of 2010 in residential facilities for older people where the unit of randomisation was the facility or a physical part of it, for example a ward, wing, or floor. We used the Medical Subject Headings definition of residential care facilities: long-term care facilities which provide supervision and assistance in activities of daily living with medical and nursing services when required. We extended this definition to other group-living arrangements where some care is provided, for example, retirement villages. The majority of the residents included in a trial had to be over the age of 60. We excluded quasi-experimental cluster designs, studies reported as ‘pilot’ , ‘feasibility’ , or ‘preliminary’ studies, studies in which no outcomes were reported, and reports on cost-effectiveness. No studies were excluded on the basis of quality, since our aim was to provide a description of quality. No trial was excluded on the basis of language or date of publication. Following the example of a previous review [24], secondary reports of individual trials were included if they reported different outcomes from the primary report.

### Data sources and search methods

We searched PubMed for relevant reports in early 2011. The full electronic search strategy is given in (Additional file 1: Box 1). KDO hand-searched the electronic archives of journals identified from initial scoping searches as those publishing at least five potentially relevant papers: the British Medical Journal, Journal of the American Medical Association, BioMed Central Health Services Research, Age and Ageing and the Journal of the American Geriatrics Society, back to the year 2000. We also conducted citation searches of each eligible report identified to find additional eligible trial reports. Finally, we contacted authors identified from the searches as having published at least three primary reports of trials in the area, asking them to identify additional studies.

### Sifting and validation

The validation process was defined in the protocol as consisting of an independent reviewer assessing the eligibility of papers that turn up in the searches strictly following the inclusion criteria without prior knowledge of the decision made by the lead reviewer as to the suitability of the paper.

To validate hand and citation searches performed by KDO, RF hand-searched the electronic version of the Journal of the American Geriatric Society for 2004, the Journal of the American Medical Association for 2000, and the references of two included papers [25,26].

KDO examined titles and abstracts of trials identified from electronic searches and obtained full texts, of those definitely or possibly satisfying inclusion criteria. To validate, RF carried out the same process on a random 10% selection of the electronically identified trials, a validation method used by previous reviews of this type [10]. When it was unclear from full texts whether a trial should be included, a final decision was made by SE.

### Data extraction

KDO extracted data from all reports; SE or RF completed a second, independent, extraction for every report. Discrepancies were resolved by discussion. We used written guidance, agreed in advance of any extractions, when extracting data. Data were extracted onto a front-end program developed by us to assist consistency of recording and field completion in the data extraction process and to transfer extracted data to a spreadsheet. We included relevant data reported in trial protocols and other publications when these were clearly referenced in the main publication.

### Data extracted

To describe the trials, we extracted data on publication date, country (or countries) in which the trial was set and one primary outcome per report, defined as that specified by the authors, or if not specified, the outcome used in sample size calculations. If no primary outcome was specified and no sample size calculation was reported, the first outcome presented in the abstract was considered primary. We divided the primary outcomes into broad categories, for example: use of restraint, falls, depression, or medication use.

To assess the quality of trials, we created a list of quality items based on the extended CONSORT statement for cluster randomised trials [13], items included in previous methodological reviews [3,4,27] and decisions by our team. See (Additional file 1: Table A).

To classify the potential for identification/recruitment bias resulting from lack of blinding of those identifying or recruiting potential participants, we used a graphical aid previously published [27], based on the definitions used by Eldridge *et al*. [4].

To investigate the effect of the strength of the journal’s endorsement of the extended CONSORT statement on quality, in early 2011, KDO and RF independently extracted all relevant text referring to the statement from the online versions of the ‘Instructions to authors’ of journals in which trial reports from 2005 onwards (after the publication of the extended CONSORT statement) were published. We adapted a previously used scale to classify journals as having high endorsement of the CONSORT statement [28,29]. If the journal website used the words ‘required’ , ‘must’ , ‘should’ , or ‘strongly encouraged’ in relation to adherence to the CONSORT checklist, medium endorsement if the website used the words ‘encouraged’ or ‘recommended’ or ‘advised’ or ‘please’ , and low endorsement if the website used words such as ‘may wish to consider’ or ‘see CONSORT’ or there was no mention of the CONSORT statement.

We used statistician co-authorship as an indication of active statistician involvement. Based on a previously used criterion [15,30], we considered the report as having a statistician (or an epidemiologist or other quantitative methodologist) as co-author if at least one of the authors listed in the report belonged to a department/unit of biostatistics/epidemiology or mathematical sciences or was clearly designated as a statistician/epidemiologist. KDO and SE independently reviewed declared affiliations and qualifications in trial reports and when these were not clearly reported searched the web for affiliations and qualifications of authors; when it was impossible to ascertain whether or not there was a statistician as co-author, this was recorded as missing. To minimise bias, we conducted these reviews separately from the main data extraction.

KDO and RF piloted the full inclusion and extraction process on a 10% sample of reports identified by electronic searches and hand searching. We refined the protocol accordingly and it is available from the authors.

### Analysis

We present descriptive statistics on trial characteristics, and describe examples of some common types of intervention. For each quality criterion, we present the overall percentage adhering to relevant recommendations. To explore reasons for variation in quality we also present these percentages by whether the trial was published in 2004 (when the first extended CONSORT statement for cluster randomised trials was published) or earlier, or after 2004; by whether the trial had statistician involvement or not; and for those trials published after 2004, by the strength of the journal’s endorsement of the CONSORT statement. To avoid difficulties in interpreting multiple comparisons in the context of a small sample size, we conducted formal statistical analyses of the relationship between quality and journal CONSORT endorsement, statistician involvement, and publication period for three key methodological quality indicators selected from the methodological literature [14,31,32] and in consultation with three experts in the field. These were ‘reported accounting for clustering in sample size calculations’ , ‘reported accounting for clustering in analysis’ and ‘low or no potential for selection bias’. They reflect the major issues affecting the validity of cluster randomised trials, as failing to account for clustering in the sample size may result in loss of power to detect statistically significant differences, not accounting for clustering in the analysis results in a higher risk of a Type I error (i.e., erroneously concluding there was a statistically significant difference), and selection bias may be present since cluster randomised trials often recruit their participants after the clusters have been randomly allocated to treatment [3,4].

We conducted univariable and multivariable logistic regression accounting for statistician co-authorship, publication period, and a variable with four categories (publication up to 2004, publication after 2004 and low CONSORT endorsement by journal, publication after 2004 and medium CONSORT endorsement, publication after 2004 and high CONSORT endorsement) that combined publication period and journal CONSORT endorsement. We conducted all analyses using Stata release 11 [33].

## Results

**Figure 1:**
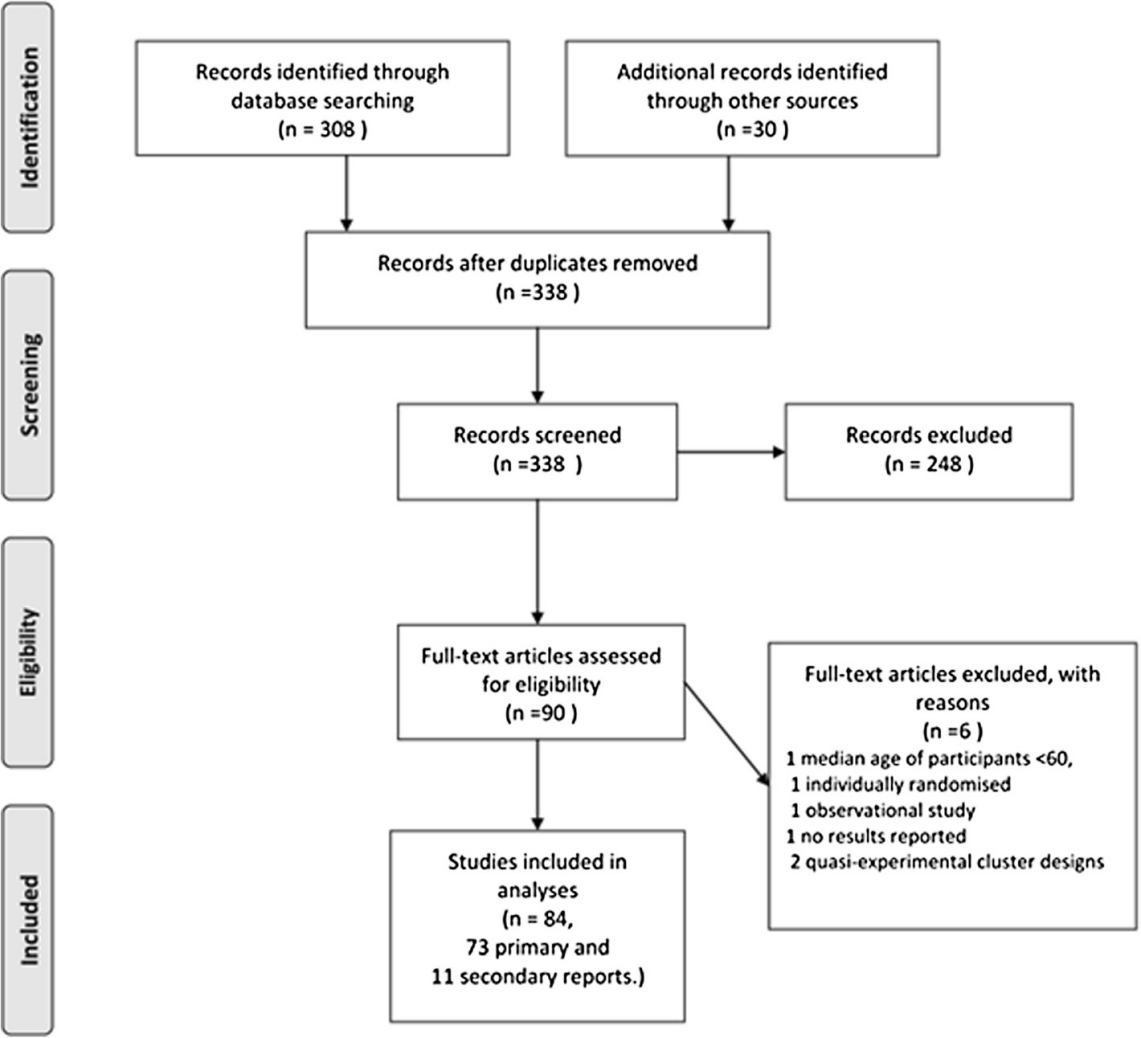
Flow diagram of the identification process for the sample of 73 cluster randomised trials included in this review.

We identified 308 published reports via our electronic search, rejecting 248 on the basis of titles or abstracts (56 non-randomised studies, 68 individually randomised trials, 95 cluster randomised trials either not in residential facilities for older people or in such facilities but randomising units other than the facility or a part of a facility, 21 protocols and other reports with no results, seven pilot or feasibility studies, one cost-effectiveness study). Additionally, 11 reports were found by hand searching (corresponding to references [A12, A14, A20, A26-A28, A45, A48, A50, A78, A83], Additional file 1), 16 by searching the references of included reports, and three from experts (references [A30, A68, A69], see Additional file 1), making a total of 90 full-text papers to be examined (Figure 1).

From these, 73 primary [A1-A73, A78] and 11 secondary reports [A74-A84] met our eligibility criteria (see Additional file 1: Tables B and C). Fifty-five had been identified by our electronic search. The full reference list is reported in the Appendix (reference numbers are preceded by an A to indicate they correspond to the reference list in the Additional file 1).

There was 93% agreement between the two reviewers for the 10% random sample used for the electronic search sifting validation (based on 31 reports), with a Cohen kappa coefficient of 0.87. The reviewers agreed on 100% of the hand-searches.

### Trial characteristics and interventions

Of the 73 included trials, over half were conducted in North America or the UK (Table 1). The number of clusters randomised ranged from three [A45] to 223 [A55] and the number of individuals included in the analysis from 41 [A3] to 6636 [A73]. There were 68 parallel trials, two with factorial designs [A38, A50], four cross-over trials [A27, A28, A45, A48], and one split-plot design where each individual acted as his or her own control [A44]; 27 trials (38%) were matched in pairs and two in triplets, 18 (24%) were stratified and the rest were randomised without any restrictions.

**Table 1:** Characteristics of trials

**Characteristic**	**Number of trials (%)**
Publication year	
1992-2004	29 (40)
2005-2010	44 (60)
Country	
USA	17 (23)
UK	16 (22)
Netherlands	9 (12)
Canada^a^	7 (10)
Australia	5 (7)
Sweden	5 (7)
Others	14 (19)
Number of clusters randomised	
Median (IQR)	15 (10, 36)
Range	3 to 230
Average cluster size^b^	
Median (IQR)	26.2 (14.8, 42.9)
Range	3.2 to 201.1
Primary focus of research (based on primary outcome)	
Falls or fractures	21 (29)
Medication use	13 (18)
Quality of Life	13 (18)
Use of restraints	6 (8)
Respiratory	4 (6)
Depression	2 (3)
Other	14 (19)

^a^ of which two jointly with the USA.

^b^ Defined as number of individuals analysed/ number of clusters analysed, and which had 17 missing values, because we couldn’t obtain either the number of individuals or of clusters in the analysis.

Interventions usually targeted staff in the facilities, residents, or both, though a few targeted premises (for example, modification of the residents’ environment). Interventions aimed at staff were typically education and training, or the provision of a tool such as a falls risk assessment tool or a computerised decision support system. Interventions aimed at residents were typically treatments such as vaccines or hip protectors, or programmes of assessment and activity which often took place over a period of time, aimed at, for example, increasing mobility. See (Additional file 1: Box 2).

### Trial quality

Quality was variable (Table 2). The word ‘cluster' was used in the title or abstract in 51/84 (61%) reports. A clustered design was explicitly justified in 30 primary reports (42%) while the intervention in two further trials involved changing the light fittings of the whole facility, making individual allocation impossible [34,35].

**Table 2:** Number (proportion) of primary reports adhering to quality criteria by key characteristics

**Criterion**		**Total**		**Publication year**				**Statistician co-authored**				**Strength of CONSORT endorsement**^**c**^					
		**N = 73**		**Before 2005**		**2005 or later**		**No**		**Yes**		**Low**		**Medium**		**High**	
								**N = 22**		**N = 51**		**N = 11**		**N = 18**		**N = 15**	
				**N = 29**		**N = 44**											
Term ‘cluster’ included in title or abstract		44	(60)	10	(34)	34	(77)	7	(32)	37	(73)	7	(64)	15	(83)	12	(80)
Cluster design justified		30	(41)	11	(38)	19	(43)	9	(41)	21	(41)	4	(36)	8	(44)	7	(47)
Eligibility criteria reported for individuals		65	(89)	25	(86)	43	(98)	20	(91)	45	(88)	9	(82)	16	(89)	15	(100)
Eligibility criteria reported for clusters		44	(60)	17	(59)	28	(64)	13	(59)	31	(61)	5	(45)	10	(56)	12	(80)
Sample size calculation reported		43	(59)	14	(48)	29	(66)	8	(36)	35	(69)	6	(55)	11	(61)	12	(80)
Clustering accounted for in sample size calculation		20	(27)	4	(14)	16	(36)	2	(9)	18	(35)	4	(36)	5	(28)	7	(47)
Evidence of variation in cluster size considered		4	(6)	0	(0)	4	(9)	1	(4)	3	(6)	1	(9)	1	(6)	2	(13)
Restricted randomisation		50	(68)	18	(62)	32	(73)	15	(68)	35	(69)	10	(91)	12	(67)	10	(67)
Potential for	None	36	(49)	14	(48)	22	(50)	12	(57)	24	(47)	7	(64)	8	(45)	7	(47)
identification/recruitment bias	Unclear	24	(32)	13	(45)	11	(25)	9	(41)	15	(29)	2	(18)	4	(22)	5	(33)
	Unlikely	8	(11)	1	(3)	7	(16)	0	(0)	8	(16)	2	(18)	4	(22)	1	(6)
	Possible	5	(7)	1	(3)	4	(9)	1	(5)	4	(8)	0	(0)	2	(11)	2	(13)
Outcome assessor blind to allocation		32	(44)	12	(41)	20	(45)	9	(41)	23	(45)	6	(55)	7	(39)	7	(47)
Clustering accounted for in analysis		54	(74)	19	(66)	35	(80)	13	(59)	41	(80)	8	(73)	15	(83)	12	(80)
Numbers of clusters randomised reported		71	(97)	29	(100)	42	(95)	21	(95)	50	(98)	9	(82)	18	(100)	15	(100)
Reported baseline of individual characteristics		69	(95)	28	(97)	41	(93)	22	(100)	47	(92)	10	(91)	17	(94)	14	(93)
Baseline of cluster characteristics reported		27	(37)	7	(24)	20	(45)	4	(18)	24	(47)	5	(45)	10	(56)	6	(40)
P-values not calculated for individual baseline comparisons		41	(56)	16	(55)	25	(57)	9	(43)	32	(63)	2	(18)	13	(72)	10	(67)
Reported numbers of clusters analysed^d^		63	(86)	25	(86)	39	(89)	18	(81)	46	(90)	9	(82)	16	(89)	14	(93)
Reported numbers of individuals analysed		66	(90)	24	(83)	39	(89)	21	(95)	42	(82)	8	(73)	17	(94)	14	(93)
Intra-cluster correlation coefficient (ICC) from analysis reported^e^		8	(14)	1	(5)	7	(20)	1	(5)	7	(18)	0	(0)	4	(14)	4	(33)
Adverse events reported		17	(23)	5	(17)	12	(27)	3	(14)	13	(25)	4	(36)	4	(22)	9	(60)

^c^ Only 44 reports; as this categorisation applies only to publications after 2004.

^d^ Data taken from results tables if not explicit.

^e^ There were 17 reports where ICC calculations were not applicable (given the aggregated nature of outcome) hence only 56 reports are considered.

Sample size calculations were described in 60% (43/73) of primary reports; clustering was accounted for in 47% (20/43) of these. Clustering was accounted for in the primary analyses of 74% (54/73) of the trials. We assessed recruitment/identification bias as ‘not possible’ in 36 trials (49%), unlikely in 8 trials (11%), unclear in 24 trials (33%), and possible in 5 trials (7%).

Many other quality criteria were reasonably well reported, but few primary reports reported intra-cluster correlation coefficients (eight trials) or mentioned adverse events (17 trials). Information about individuals was better reported than information about clusters. For example, loss to follow-up of individuals was described in 53 reports but loss to follow-up of clusters in only 28. Blinding of primary outcome assessors was clearly reported in only 32 (44%) trials.

In extracting data, we identified further quality issues in relation to the design of some trials. For example, authors of four trials reported that contamination between intervention arms may have occurred or indeed had occurred, due to the randomisation of wards or floors within the same residential unit to different intervention arms [A53, A57, A72, A82]. Since one rationale for cluster randomisation is to prevent contamination, its occurrence within such trials suggests lack of adequate planning at the design stage.

### Determinants of quality

We included 44 primary reports published since 2005 in 22 journals, 15 in journals strongly endorsing CONSORT, 18 in journals with medium endorsement and 11 in journals with low endorsement (See (Additional file 1: Table D) for details). Of the 73 primary reports, 51 included a statistician or other quantitative methodologist as a co-author. Statistician co-authorship and publication period appeared to have stronger influences on quality than strength of a journal’s endorsement of the CONSORT statement (Table 2).

In univariable analyses (Table 3), trials with statisticians as co-authors were more likely to account for clustering in the analysis (unadjusted OR 3.2, 95% confidence interval (CI) 1.2 to 8.5) and sample size calculation (OR 5.4, 95% CI 1.1 to 26.0), but evidence for the effect of statistician co-authorship on identification and recruitment bias was inconclusive (OR 1.4, 95% CI 0.5 to 3.8). Trials published after 2004 were more likely to account for clustering in the sample size (unadjusted OR 3.6 95% CI 1.1 to 12.1) but although the direction of estimates was consistent, there was no evidence that publication period affected the likelihood of either accounting for clustering in the analysis (unadjusted OR 1.3 95% CI 0.5 to 3.2) or identification/recruitment bias (unadjusted OR 1.9 95% CI 0.7 to 4.7). There appeared to be little effect of journal endorsement of the extended CONSORT statement on any of the methodological quality markers. Few effects were statistically significant (at 5% level) in multivariable analyses (Table 3).

**Table 3:** Unadjusted and adjusted odds ratio of adhering to quality criteria

	**Proportion adhering to criteria**	**Unadjusted odds ratio**		**Adjusted**^**g**^**odds ratio**	
Reported accounting for clustering in analysis (includes secondary reports, 84 reports)					
No statistician co-author	16/28	Reference category			
Co-author included statistician	44/56	3.2	(1.2,8.5)	3.1	(1.1,8.7)
Publication prior to 2005	22/33	Reference category			
Journal CONSORT endorsement low^f^	9/14	0.9	(0.2,3.3)	0.8	(0.2,3.3)
Journal CONSORT endorsement medium^f^	16/21	1.6	(0.5,5.5)	1.1	(0.3,4.2)
Journal CONSORT endorsement high^f^	12/16	1.5	(0.4,5.8)	1.4	(0.3,6.5)
Reported accounting for clustering in sample size (unique trials, 73 reports)					
No statistician co-author	2/22	Reference category			
Co-author included statistician	18/51	5.4	(1.1,26.7)	2.7	(0.5,16.2)
Publication prior to 2005	4/29	Reference category			
Journal CONSORT endorsement low^f^	4/11	3.6	(0.7,18.0)	3.5	(0.6,18.8)
Journal CONSORT endorsement medium^f^	5/18	2.4	(0.5,10.5)	1.7	(0.4,7.7)
Journal CONSORT endorsement high^f^	7/15	5.5	(1.3,23.6)	4.7	(1.0,21.8)
Identification/recruitment bias is not possible or unlikely (unique trials, 73 reports)					
No statistician co-author	12/22	Reference category			
Co-author included statistician	32/51	1.4	(0.5,3.9)	1.3	(0.4,4.0)
Publication prior to 2005	15/29	Reference category			
Journal CONSORT endorsement low^f^	9/11	4.2	(0.8,22.9)	4.1	(0.8,22.7)
Journal CONSORT endorsement medium^f^	12/18	1.9	(0.5,6.3)	1.8	(0.5,6.4)
Journal CONSORT endorsement high^f^	8/15	1.1	(0.3,3.7)	10.2	(0.3,4.5)

^f^ Reference category is publication pre-2005.

^g^ Adjusted for the other variables in the table.

## Discussion

This first review of cluster randomised trials in residential facilities for older people shows that there is a large number of cluster randomised trials in this field and that their quality, although variable, has improved over time. Reporting of sample size calculations, processes for identification and recruitment of individual residents, blinding of outcome assessors, adverse events, intra-cluster correlation coefficient, and cluster characteristics appear to have been particularly poor. There is stronger evidence of the effect of the presence of a statistician (or other quantitative methodologist) amongst co-authors, than of the strength of a journal’s endorsement of CONSORT recommendations on the methodological quality of the trials.

### Strengths and limitations

We used rigorous searching and data extraction procedures including focused hand-searching resulting in a comprehensive overview of cluster randomised trials in residential facilities for older people. That almost one-third of trials were not found using our electronic search probably reflects poor reporting. Although our search strategy compares favourably with one recently reported with excellent sensitivity [36], we are aware that we may have missed some cluster randomised trials in nursing home settings, as the hand-searches were performed in only five journals and hand searching provided a third of our sample.

The size of our sample may be too small to detect some associations, and results must be interpreted accordingly. Our methods for identifying whether a statistician was part of the writing team extended those previously used [15,30], by incorporating a web-search of all authors in the report. A possible limitation of our study is that journal instructions may not have been the same when trial reports were actually published (between 2005 and 2010) as they were when we extracted information on journals’ endorsements of the extended CONSORT statement in 2011.

Our assessment of quality was based only on information in trial reports. Space limitations in journals may sometimes preclude reporting of some factors relevant to quality in published reports, although this may change with increasing on-line publishing. Although there is no stated consensus on the most important quality items, the quality criteria we used are largely a subset of the CONSORT checklist, and we sought expert opinion before choosing the specific methodological quality items to formally analyse. The updated extension to the CONSORT statement for cluster randomised trials [37], which was published after we completed this review, acknowledges the importance of considering two issues we include in this review: the effect of variable cluster size on sample size calculations and expands on item 10 to recommend specific reporting of the way in which individuals are included in the trial.

A further possible limitation to our conclusion is the choice of the year 2005 to dichotomise publication year, and its use in analyses corresponding to the CONSORT statement impact on quality. We recognise that the extended CONSORT guidelines were published in 2004 and that, due to time lags between submission of a report and publication, a few of the reports we analysed may not have been aware of these guidelines. However, the guidelines were in development and draft versions of them were common knowledge a few years prior to their final appearance in print, so we consider this may not be a severe limitation on validity.

Finally, authors may fail to report that their trials are cluster-randomized; we may therefore have missed some trials due to this.

### Comparison with previous research

Proportions of trials in which clustering was accounted for in the sample size and analysis (Table 4) were similar to those for cluster randomised trials in all fields [12], and in the fields of tropical disease and oncology [11,38], but lower than in other areas [4,24,27]. Relative to previous reviews, we judged that identification/recruitment bias was possible in a lower percentage (7%) and unclear in a higher percentage (32%) of our included trials [4,27].

**Table 4:** Percentages accounting for clustering in sample size calculations and analysis in previous reviews

**Authors**	**Source of trials**	**Years**	**Clustering accounted for**	
			**In sample size**	**In analysis**
Donner et al. [5]	16 non-therapeutic Intervention trials	1979-1989	19%	50%
Simpson et al. [6]	21 trials from Amer J Pub Health Prev Med	1990-1993	19%	57%
Chuang et al. [7]	24 trials of computer based decision support	1975-1998	0%	58%
Isaakidis et al. [8]	51 trials in Sub-Saharan Africa	1973-2001	20%	37%
Puffer et al. [3]	36 trials in BMJ, Lancet and NEJM	1997-2002	56%	92%
Eldridge et al. [10]	152 trials in primary health care	1997-2000	9%	59%
Varnell et al. [39]	60 trials in Amer J PubHealth Prev Med	1998-2002	15%	54%
Bland [9]	18 articles in BMJ	1983-2003	n/a	72%
Eldridge et al. [4]	34 articles in primary health care	2004-2005	62%	88%
Murray et al. [38]	75 trials in oncology	2002-2006	24%	45%
Bowater et al. [11]	35 trials in tropical parasitic diseases	1998-2007	29%	43%
Handlos et al. [24]	35 trials in maternal and child health	1998-2008	71%	80%
Froud et al. [27]	23 trials in oral health	2005-2009	65%	78%
Ivers et al. [12]	300 trials of cluster design randomly selected	2000-2008	33%^h^	70%
This review	73 trials in residential facilities for older people	1993-2010	27% ^i^	74%

^h^ Calculated by us for consistency with other reports, as the reported 61% excludes those trials which did not report sample size calculations.

^i^ This increases to 47% when those that did not report sample size calculations are excluded.

Ours is not the first review to identify particularly poor reporting of factors such as observed intra-cluster correlation coefficients and cluster characteristics that have less influence on the validity of the main trial analyses than on their usefulness to practitioners, policy makers and future researchers [4,10,12]. Overall, proportions adhering to various criteria in our review were not dissimilar to proportions in the recent review of cluster randomised trials in all fields that used a similar set of quality criteria [40]. The effect of the presence of a statistician in the research team on study quality supports previous research in the context of individually randomised trials [15]. In contrast, we found no evidence of an association between journal endorsement of the 2004 extended CONSORT statement and reporting quality, contrary to the findings of previous research, which identified better reporting amongst randomised controlled trials published in journals more strongly promoting the original 1996 CONSORT statement [16].

### Interpretation

Our review covers a longer time period than some other recent reviews of cluster randomised trials. Twentieth century trials included in our review tended to be lower quality with, for example, none accounting for clustering in the sample size calculation. This may explain why our results suggest an improvement over time in this criterion when the review of cluster randomised trials in all fields between 2000 and 2008 did not [12]. A further explanation is that trends in one field may be masked when trials from all fields are reviewed, particularly in a research area such as cluster randomised trials in which the number of studies is growing. Although the numbers of trials included in our analyses preclude definitive conclusions, we hypothesise that improvements in reporting of these trials have been taking place gradually since the turn of the millennium when there was a proliferation of books and journal articles relating to cluster randomised trials, but at different rates in different fields, and the extended CONSORT statement has been a reinforcement of that change rather than a precipitator. This is consistent with similar phenomena of procedural or organisational change taking place *after* individual behaviour change seen in other contexts in society at large.

While published guidelines may have been successful in effecting some change in reporting practices, it is plausible that the presence of suitably qualified individuals on a research team exerts a greater influence on trial design, conduct and analysis than written instructions to adhere to such guidelines. The latter may influence reporting more than they influence trial design. Although we only explored the impact of the presence of a statistician on three key methodological features, we acknowledge that individuals with other types of expertise may have a similar effect on key aspects of trials in other areas.

## Conclusions

Cluster randomised trials in residential facilities need improvement. Direct statistician involvement may improve the methodological quality of cluster randomised trials more than journal endorsement of the CONSORT statement. Investigators should always involve a statistician in their trial team and funders and journals should encourage this.

## Abbreviations

CRTs: Cluster randomised trials; CONSORT: Consolidated standards of reporting trials; ICC: Intra-cluster correlation coefficient.

## Competing interest

Two of the authors are statisticians (KDO and SE).

## Authors’ contributions

SE conceived the study, BS advised on the design of the study and contributed to the protocol, KDO contributed to the design of the study, wrote the protocol and designed the extraction interface, based on one previously designed by RF. KDO, RF and SE undertook data extraction. KDO conducted the analyses of the data. All authors had full access to all the data. KDO and SE took primary responsibility for writing the manuscript. All authors provided feedback on all versions of the paper. All authors read and approved the final manuscript.

## Pre-publication history

The pre-publication history for this paper can be accessed here:

http://www.biomedcentral.com/1471-2288/13/127/prepub

## Supplementary Material

Additional file 1 **Box 1.** Electronic search strategy, **Table A** Quality criteria and other data extracted from publications, **Table B** and **C**. Characteristics of included studies, **Box 2** Examples of interventions and **Table D** CONSORT endorsement by journals included in this review. Full bibliography of the studies included in this review. Click here for file
