# Supplementary material for: A systematic review of cluster randomised trials in residential facilities for older people suggests how to improve quality
Source: BMC Med Res Methodol. 2013 Oct 22;13:127. doi: 10.1186/1471-2288-13-127 (PMC4015673; doi:10.1186/1471-2288-13-127)
Supplement: Additional file 1 — Box 1. Electronic search strategy, Table A Quality criteria and other data extracted from publications, Table B and C. Characteristics of included studies, Box 2 Examples of interventions and Table D CONSORT endorsement by journals included in this review. Full bibliography of the studies included in this review. [file 1471-2288-13-127-S1.pdf]

Additional file 1

Box 1 Search strategy: Our full identification strategy optimised for PubMed .

#1: (randomized trial) AND (clinical trial) [All Fields]

#2: ((long term care facility) OR (long term care) OR (assisted living) OR (group homes) OR (homes of aged) OR (Residential facilities) OR (nursing home) OR (retirement homes) OR (retirement communities)) [All Fields]

#3: ((cluster randomization) OR (cluster randomisation) OR (cluster) OR (clustered) OR (clustering) OR (clusters) OR (group-randomized) OR (group-randomised) OR (randomisation unit) OR (randomization unit)) [All Fields]

#4: #1 AND #2 AND #3

Table A. Quality criteria and other data extracted from publications

Quality criteria	Data extracted (mostly yes/no)	Source[i]
Term 'cluster' included in title or abstract	Was the term 'cluster' included in title or abstract?	Item 1
Cluster design justified	Reasons for choosing cluster randomisation given?	Item 2
Design type specified	Parallel, factorial, cross-over, other	Team (3a)
Reported eligibility criteria for participants	Reported eligibility criteria for participants?	Item 3 (4)
Reported eligibility criteria for clusters	Reported eligibility criteria for clusters?	Item 3 (4)
Sample size calculation reported	Was a sample size calculation reported?	Item 7 (7a)
Clustering accounted for in sample size calculation	Use of intra-cluster correlation coefficient, k or design effect to allow for clustering?	Item 7 (7a)
Evidence of variation in cluster size considered	Increased sample size to account for this (any method acceptable)?	Team decision
Reported method used to generate random allocation sequence	Simple randomisation, stratification, blocking, matching, minimisation	Item 8 (8b)
Minimised identification/ recruitment bias	Were participants identified/recruited prior to randomisation, or were recruiters blinded to allocation?	Item 10[k]
Reported on blinding of outcome assessors	Were outcome assessors (for primary outcome) reported as blind to assessment? (Note: if it was not applicable, e.g. recorded from routine data, the question was answered as 'Yes')	Item 11[k]
Clustering accounted for in analysis	Did the report describe an analysis that accounts for clustering?	Item 12a
Reported number of clusters randomised	Number of clusters randomised in each arm	Item 13a
Reported number of individuals randomised	Number of individuals randomised in each arm	Item 13a
Baseline characteristics of clusters reported	Was there a table or text indicating cluster-level baseline comparison between intervention and control?	Item 15
Baseline characteristics of individuals reported	Was there a table or text indicating individual-level baseline comparison between intervention and control?	Item 15
No statistical significance tests on baseline data	P-values not calculated for baseline comparisons of individual participant characteristics?	Previous review
Reported number of clusters analysed	Number of clusters in each arm for analysis of primary outcome	Item 16
Reported number of individuals analysed	Number of individuals in each arm for analysis of primary outcome	Item 16
ICC reported	ICC reported for at least one outcome?	Item 17
Adverse events reported	Number of adverse events (or statement that none) reported	Item 19
**Other information**	**Data extracted**	**Source**
Statistician involvement	Was a statistician, epidemiologist or other quantitative researcher a co-author in the report?	Team decision
Primary outcome measure	as designated by the authors, or the one used in sample size calculation, or the first outcome reported in the abstract	Team decision

[i] Items refer to 2004 extended CONSORT statement; numbers in parentheses are CONSORT items from the updated 2010 main CONSORT statement.

[k] We extracted more detail than simply whether or not the recruiter/identifier of participants was blind to allocation status, see Figure 1 for decision-aid.

Table B Basic characteristics of primary reports included in review

Author	Year	Journal	Country	Outcome measure	Individuals analysed*	Clusters randomised*
Avorn[1]	1992	N Engl J Med	USA	psychoactive drug use	678	12
Rimer[2]	1992	Public Health Rep	USA	use of mammograms	412	8
McMurdo[3]	1993	Age and Ageing	UK	battery of physical measures	41	4
Evans[4]	1997	J Am Geriatr Soc	USA	use of physical restraints	463	16
Meador[5]	1997	J Am Geriatr Soc	USA	antipsychotic use	1152	12
Ray[6]	1997	JAMA	USA	recurrent fallers	482	14
Schmidt[7]	1998	J Am Geriatr Soc	Sweden	psychotropic drugs use	1805	36
Morris[8]	1999	J Gerontol A Biol Sci Med Sci	USA	activities of daily life summary scale	392	6
Proctor[9]	1999	Lancet	UK	Crichton Royal Behaviour rating scale	105	12
McMurdo[10]	2000	Gerontology	UK	number of residents sustaining a fall	133	9
Furniss[11]	2000	Br J Psychiatry	UK	Crichton Royal Behaviour rating scale	294	14
Molloy[12]	2000	JAMA	Canada	residents' and families' satisfaction with health care and health care services utilization	n/r	6
Roberts[13]	2001	Br J Clin Pharmacol	Australia	continuous drug use	n/r	52
Naughton[14]	2001	J Am Geriatr Soc	USA	antibiotic use	n/a	10
Frenkel[15]	2001	Community Dent Oral Epidemiol	UK	denture plaque scores	378	22
Jensen[16]	2002	Ann Intern Med	Sweden	number of residents sustaining a fall	384	9
Midlov[17]	2002	Eur J Clin Pharmacol	Sweden	Short Form-36	n/r	48
Lee[18]	2002	J Am Geriatr Soc	Hong Kong	Barthel index score	89	45
Lord[19]	2003	J Am Geriatr Soc	Australia	falls	508	20
Wolf[20]	2003	J Am Geriatr Soc	USA	falls	286	20
Becker[21]	2003	J Am Geriatr Soc	Germany	rate of falls	981	6
Meyer[22]	2003	BMJ	Germany	hip fracture	942	49
O'Halloran[23]	2004	Age and Ageing	Northern Ireland	fractured proximal femur	n/a	127
Crotty[24]	2004	Age and Ageing	Australia	Medication Appropriateness Index	n/r	10
Dyer[25]	2004	Age and Ageing	UK	number of falls per person	196	20
Crotty[26]	2004	BMC Health Serv Res	Australia	fall rate	715	20
Gold[27]	2004	J Am Geriatr Soc	USA	trunk extension strength	122	9
Sloane[28]	2004	J Am Geriatr Soc	USA	care recipient behaviour assessment	69	15
Kerse[29]	2004	J Am Geriatr Soc	New Zealand	proportion of residents sustaining a fall	547	14

Author	Year	Journal	Country	Outcome measure	Individuals analysed*	Clusters randomised*
Eisses[30]	2005	Br J Psychiatry	Netherlands	geriatric depression scale GDS-15	360	10
Loeb[31]	2005	BMJ	Canada and USA	antimicrobials prescription	3754	24
Ray[32]	2005	Arch Intern Med	USA	serious fall-related injuries	n/r	112
Bravo[33]	2005	Int J Qual Health Care	Canada	quality of care scores (QUALCARE Scale)	122	40
Testad[34]	2005	Int J Geriatr Psychiatry	Norway	use of physical restraints	142	4
Law[35]	2006	Age and Ageing	UK	incidence of falls	3717	223
Hayward[36]	2006	BMJ	UK	all-cause mortality	2572	50
Nijs[37]	2006	BMJ	Netherlands	quality of life using validated questionnaire	178	10
Rosendahl[38]	2006	Aust J Physiother	Sweden	Berg Balance scale.	172	34
Huizing[39]	2006	BMC Geriatr	Netherlands	use of physical restraints	126	5
Loeb[40]	2006	JAMA	Canada	hospital admissions rates	661	22
Fossey[41]	2006	BMJ	UK	neuroleptics prescription	346	12
Monette[42]	2007	J Am Geriatr Soc	Canada	adherence to the recommendations	n/r	10
Kovacs[43]	2007	Spine (Phila Pa 1976)	Spain	Roland-Morris questionnaire	584	12
Kiel[44]	2007	JAMA	USA	adjudicated hip fractures	1042	37
Sloane[45]	2007	J Am Geriatr Soc	USA	night-time sleep using wrist actigraphy and daytime activity using non-obtrusive daytime observations	66	3
Colon-Emeric[46]	2007	Am J Med	USA	prescription of osteoporosis pharmacotherapy or hip protectors	n/r	67
Orrell[47]	2007	Int J Geriatr Psychiatry	UK	Camberwell Assessment of Needs in Elderly	192	24
Simmons[48]	2008	J Am Geriatr Soc	USA	residents' weight	76	4
Gurwitz[49]	2008	J Am Geriatr Soc	USA and Canada	adverse drug events	1118	29
Riemersma-van der Lek[50]	2008	JAMA	Netherlands	Mini Mental State Examination	189	12
Kerse[51]	2008	BMJ	New Zealand	elderly mobility scale and time to fall.	473	41
Bouwen[52]	2008	Age and Ageing	Belgium	number of participants with at least one accidental fall requiring an intervention by a physician or a nurse	379	10
Peri[53]	2008	Age and Ageing	New Zealand	Short Form-36	n/r	10
Bentzen[54]	2008	Osteoporos Int	Norway	adherence between people offered soft and hard hip protectors	n/r	18

**Author**	**Year**	**Journal**	**Country**	**Outcome measure**	**Individuals analysed***	**Clusters randomised***
Cox[55]	2008	Age and Ageing	UK	hip fractures	5637	230
Gopal Rao[56]	2009	Epidemiol Infect	UK	proportion of compliance with infection control guidelines	n/a	12
Field[57]	2009	J Am Med Inform Assoc	Canada	appropriate drug orders within alert categories	833	22
Huizing[58]	2009	J Am Geriatr Soc	Netherlands	use of physical restraints	241	15
Kuske[59]	2009	Int Psychogeriatr	Germany	GEROLF - staff knowldege in dealing with dementia	210	6
Lemaitre[60]	2009	J Am Geriatr Soc	France	all-cause mortality	3400	40
Resnick[61]	2009	J Am Geriatr Soc	USA	Barthel index score	n/r	12
Meyer[62]	2009	Age and Ageing	Germany	number of residents sustaining a fall	1125	58
Patterson[63]	2009	J Am Geriatr Soc	Northern Ireland	inappropriate psycho- active prescriptions	253	22
Sackley[64]	2009	BMJ	UK	Barthel index score	243	24
Eggermont[65]	2009	Behav Brain Res	Netherlands	neuropsychological tests, mood questionnaires and actigraphy	61	n/a
Neyens[66]	2009	Age and Ageing	Netherlands	falls	518	12
Marcantonio[67]	2010	J Am Geriatr Soc	USA	persistence of delirium	370	8
Testad[68]	2010	J Clin Psychiatry	Norway	Cohen-Mansfield Agitation Inventory	145	n/r
Pellfolk[69]	2010	J Am Geriatr Soc	Sweden	use of physical restraints	288	40
Ward[70]	2010	Med J Aust	Australia	fracture neck of femur	5391	88
Baldwin[71]	2010	J Hosp Infect	Northern Ireland	*methicillin-resistant staphylococcus aureus* prevalence in residents and staff	478	32
van Gaal[72]	2010	Int J Nurs Stud	Netherlands	score on a knowledge test regarding 3 topics: pressure ulcers, urinary infection, falls	102	10
Looijmans-van den Akker[73]	2010	Vaccine	Netherlands	proportion of health care workers vaccinated against influenza	6636	36

* n/a not applicable; n/r not reported.

Table C Basic characteristics of secondary reports included in review

Author	Publication year	Related to reference	Journal	Primary outcome measure for this publication
Jensen[74]	2003	Jensen[16]	J Am Geriatr Soc	time to first fall
Warnke[75]	2004	Meyer[22]	Z Gerontol Geriatr	quality of life assessment: fear of falling
Warnke[76]	2004	Meyer[22]	J Am Geriatr Soc	time to first fall without hip protector
Jensen[77]	2004	Jensen[16]	Aging Clin Exp Res	functional ambulation categories
Sattin[78]	2005	Wolf[20]	J Am Geriatr Soc	Activities-Specific Balance Confidence Scale and the Fall Efficacy Scale
Bentzen[79]	2008	Bentzen [54]	Inj Prev	hip fractures
Rapp[80]	2008	Becker[21]	J Am Geriatr Soc	time to first fall
Rosendahl[81]	2008	Rosendahl[38]	Aging Clin Exp Res	fall rate
Huizing[82]	2009	Huizing [58]	Int J Nurs Stud	use of physical restraints
Littbrand[83]	2009	Rosendahl[38]	J Am Geriatr Soc	Barthel index score
Conradsson[84]	2010	Rosendahl[38]	Aging Ment Health	geriatric depression scale GDS-15

Box 2 Examples of interventions

Example 1: Activity for residents plus staff training (Sackley et al, 2009[64])

Activity for residents: Three-month physiotherapy and occupational therapy aimed at enhancing mobility and ability to perform activities of daily living, addressing components such as strength, flexibility, balance, and exercise tolerance, delivered by two qualified physiotherapists. Dose, frequency and duration were dependent on goals agreed by the individual participant and the therapists and on progress throughout the intervention.

Staff training: Programme aiming to promote residents' independence and use of therapeutic aids.

Example 2: Drug or technology aimed at individual residents (Law et al, 2006[35])

Ergocalciferol 2.5 mg every three months to prevent falls and fractures

Example 3: Staff training plus risk assessment tool use (Cox et al, 2008[55])

Staff training: Half-day training by specialist osteoporosis nurses organised for managers, nurses and health care assistants at central locations, aiming to support staff to recognise the importance of falls and fracture prevention and identify those at high risk.

Risk assessment tool use: The care home staff were trained to use the Black fracture risk assessment tool and the STRATIFY risk assessment tool. They used these on each resident and reported results to the specialist nurses who fed back to the care home staff and the resident's GP with an assessment of fracture and falls risk.

Example 4: Falls risk assessment tool plus information (Kerse et al, 2004[29])

Risk assessment: Each home appointed a falls coordinator to undertake risk assessment of all residents using an evidence-based falls risk assessment tool. High-risk residents had a logo attached to wall of their room.

Information: This was provided on specific fall-prevention strategies and environmental assessment of surroundings and hazards. In addition, all useful material was included in a manual.

Table D. CONSORT endorsement by journals included in this review

Journal	**Strength of CONSORT endorsement**	**Quote from 'Instructions for authors'**
Int J Geriatr Psychiatry	Low	No mention of CONSORT guidelines
Behav Brain Res	Low	No reference found in guidelines for authors
Am J Med	Low	No mention of CONSORT in guidelines for authors
J Am Medical Informatics Assoc	Low	No mention of CONSORT in guidelines for authors
Osteoporos Int	Low	No mention of CONSORT in instructions for authors
Int J Qual Health Care	Low	No mention of CONSORT in author guidelines
Aging Clin Exp Res	Low	No mention of CONSORT in instruction for authors
Aging Ment Health	Low	No mention of CONSORT in instruction for authors
Vaccine	Low	Please refer to the CONSORT statement website at http://www.consort-statement.org for more information.
Epidemiol Infect	Low	The requirements of the journal are in accordance with the International Committee of Medical Journal Editors Uniform Requirements for Manuscripts submitted to biomedical journals [*British Medical Journal* 1991; 302: 338–341 and *New England Journal of Medicine* 1991; 324: 424–428.].
Age and Ageing	Medium	For reporting of randomised trials, authors are advised to work to the guidelines in the CONSORT statement. www.consort-statement.org
JAGS	Medium	To improve the quality of reporting randomized, controlled trials (RCTs), it is recommended that authors adhere to the CONSORT (Consolidated Standards of Reporting Trials) statement, which consists of a checklist and flow diagram that authors can use to report RCTs. Authors should refer to the paper, Altman DG, et al. The revised CONSORT statement for reporting randomized trials: Explanation and elaboration. *Annals of Internal Medicine* 2001; 134:663-694
BMC Geriatrics	Medium	(the journal) also supports initiatives aimed at improving the reporting of biomedical research. Checklists have been developed for a number of study designs, including randomized controlled trials (CONSORT). Authors are requested to make use of these when drafting their manuscript and peer reviewers will also be asked to refer to these checklists when evaluating these studies.
Br J Psychiatry	Medium	The *Journal* recommends to authors the CONSORT guidelines (1996, *Journal of the American Medical Association*, 276, 637-639) and their basis (2001, *Annals of Internal Medicine*, 134, 663-694) in relation to the reporting of randomised controlled clinical trials; also recommended is their extension to cluster randomised controlled trials (2004, *BMJ*, 328, 702-708).
Archives Intern Med	High	Preparing Reports of Randomized Controlled Trials:<br>The CONSORT Checklist should be completed and submitted with the manuscript. In addition, a flow diagram illustrating the progress of patients throughout the trial should be included as a figure in the manuscript (see Figure for example). The checklist and flow diagram will be reviewed along with the manuscript.
Australian Journal Physiotherapy	High	Instruction for authors: Submit a CONSORT-like figure outlining the flow of participants through the trial, including reasons for dropouts<br>Instruction for reviewers: Reviewers are asked to consult checklists where appropriate. Specifically, reviewers of randomised controlled trials are asked to consult the CONSORT e-checklist,
BMJ	High	If you are submitting a RCT please send with your manuscript a completed checklist and flowchart in accordance to the appropriate CONSORT guidelines, the trial protocol and the registration details of the trial.
JAMA	High	All randomized clinical trials should include a flow diagram and authors should

		provide a completed randomized trial checklist (see CONSORT Flow Diagram and Checklist) and a trial protocol.
Int Psychogeriatr	High	All manuscripts reporting randomized controlled trials should have the following sent with them or they will be returned to the authors: A check list and flow chart in accordance with the CONSORT guidelines which can be found at http://www.consort-statement.org. Please send in the checklist as a supplementary file and include the flow chart as Figure 1 in the manuscript
Med J Aust	High	RCTs must follow CONSORT guidelines (including checklist and flowchart, see www.consort-statement.org).
Int J Nurs Stud	High	The editors require that manuscripts adhere to recognized reporting guidelines relevant to the research design used. These identify matters that should be addressed in your paper. These are not quality assessment frameworks and your study need not meet all the criteria implied in the reporting guideline to be worthy of publication in the journal. The checklists do identify essential matters that should be considered and reported upon. For example, a controlled trial may or may not be blinded but it is important that the paper identifies whether or not participants, clinicians and outcome assessors were aware of treatment assignments. You are encouraged (although not required) to submit a checklist from the appropriate reporting guideline together with your paper as a guide to the editors and reviewers of your paper. *Randomised (and quasi-randomised) controlled trial* - CONSORT http://www.equator-network.org/index.aspx?o=1032
Spine	High	For Clinical Trials and similar study designs, please adhere to the CONSORT statement (www.consort-statement.org/).
Injury Prev	High	Please note that we require registration of clinical trials and reporting of randomized clinical trials according to the CONSORT guidelines. We also ask that authors follow the STROBE guidelines when reporting observational studies. Although not required for publication in Injury Prevention, authors are encouraged to review similar published guidelines for the reporting of systematic reviews, meta-analyses, and assessments of diagnostic test. Templates for CONSORT, STROBE and other reporting guidelines are available through the Equator Network (http://www.equator-network.org/)

List of studies included in this systematic review

1. Avorn J, Soumerai SB, Everitt DE, Ross-Degnan D, Beers MH, Sherman D, Salem-Schatz SR, Fields D: **A randomized trial of a program to reduce the use of psychoactive drugs in nursing homes**. *N Engl J Med* 1992, **327**(3):168-173.
2. Rimer BK, Resch N, King E, Ross E, Lerman C, Boyce A, Kessler H, Engstrom PF: **Multistrategy health education program to increase mammography use among women ages 65 and older**. *Public Health Rep* 1992, **107**(4):369-380.
3. McMurdo ME, Rennie L: **A controlled trial of exercise by residents of old people's homes**. *Age Ageing* 1993, **22**(1):11-15.
4. Evans LK, Strumpf NE, Allen-Taylor SL, Capezuti E, Maislin G, Jacobsen B: **A clinical trial to reduce restraints in nursing homes**. *J Am Geriatr Soc* 1997, **45**(6):675-681.
5. Meador KG, Taylor JA, Thapa PB, Fought RL, Ray WA: **Predictors of antipsychotic withdrawal or dose reduction in a randomized controlled trial of provider education**. *J Am Geriatr Soc* 1997, **45**(2):207-210.

6. Ray WA, Taylor JA, Meador KG, Thapa PB, Brown AK, Kajihara HK, Davis C, Gideon P, Griffin MR: **A randomized trial of a consultation service to reduce falls in nursing homes**. *JAMA* 1997, **278**(7):557-562.
7. Schmidt I, Claesson CB, Westerholm B, Nilsson LG, Svarstad BL: **The impact of regular multidisciplinary team interventions on psychotropic prescribing in Swedish nursing homes**. *J Am Geriatr Soc* 1998, **46**(1):77-82.
8. Morris JN, Fiatarone M, Kiely DK, Belleville-Taylor P, Murphy K, Littlehale S, Ooi WL, O'Neill E, Doyle N: **Nursing rehabilitation and exercise strategies in the nursing home**. *J Gerontol A Biol Sci Med Sci* 1999, **54**(10):M494-500.
9. Proctor R, Burns A, Powell HS, Tarrier N, Faragher B, Richardson G, Davies L, South B: **Behavioural management in nursing and residential homes: a randomised controlled trial**. *Lancet* 1999, **354**(9172):26-29.
10. McMurdo ME, Millar AM, Daly F: **A randomized controlled trial of fall prevention strategies in old peoples' homes**. *Gerontology* 2000, **46**(2):83-87.
11. Furniss L, Burns A, Craig SK, Scobie S, Cooke J, Faragher B: **Effects of a pharmacist's medication review in nursing homes. Randomised controlled trial**. *Br J Psychiatry* 2000, **176**:563-567.
12. Molloy DW, Guyatt GH, Russo R, Goeree R, O'Brien BJ, Bedard M, Willan A, Watson J, Patterson C, Harrison C *et al*: **Systematic implementation of an advance directive program in nursing homes: a randomized controlled trial**. *JAMA* 2000, **283**(11):1437-1444.
13. Roberts MS, Stokes JA, King MA, Lynne TA, Purdie DM, Glasziou PP, Wilson DA, McCarthy ST, Brooks GE, de Looze FJ *et al*: **Outcomes of a randomized controlled trial of a clinical pharmacy intervention in 52 nursing homes**. *Br J Clin Pharmacol* 2001, **51**(3):257-265.
14. Naughton BJ, Mylotte JM, Ramadan F, Karuza J, Priore RL: **Antibiotic use, hospital admissions, and mortality before and after implementing guidelines for nursing home-acquired pneumonia**. *J Am Geriatr Soc* 2001, **49**(8):1020-1024.
15. Frenkel H, Harvey I, Newcombe RG: **Improving oral health in institutionalised elderly people by educating caregivers: a randomised controlled trial**. *Community Dent Oral Epidemiol* 2001, **29**(4):289-297.
16. Jensen J, Lundin-Olsson L, Nyberg L, Gustafson Y: **Fall and injury prevention in older people living in residential care facilities. A cluster randomized trial**. *Ann Intern Med* 2002, **136**(10):733-741.
17. Midlov P, Bondesson A, Eriksson T, Petersson J, Minthon L, Hoglund P: **Descriptive study and pharmacotherapeutic intervention in patients with epilepsy or Parkinson's disease at nursing homes in southern Sweden**. *Eur J Clin Pharmacol* 2002, **57**(12):903-910.
18. Lee DT, Lee IF, Mackenzie AE, Ho RN: **Effects of a care protocol on care outcomes in older nursing home patients with chronic obstructive pulmonary disease**. *J Am Geriatr Soc* 2002, **50**(5):870-876.
19. Lord SR, Castell S, Corcoran J, Dayhew J, Matters B, Shan A, Williams P: **The effect of group exercise on physical functioning and falls in frail older people living in retirement villages: a randomized, controlled trial**. *J Am Geriatr Soc* 2003, **51**(12):1685-1692.

20. Wolf SL, Sattin RW, Kutner M, O'Grady M, Greenspan AI, Gregor RJ: **Intense tai chi exercise training and fall occurrences in older, transitionally frail adults: a randomized, controlled trial**. *J Am Geriatr Soc* 2003, **51**(12):1693-1701.
21. Becker C, Kron M, Lindemann U, Sturm E, Eichner B, Walter-Jung B, Nikolaus T: **Effectiveness of a multifaceted intervention on falls in nursing home residents**. *J Am Geriatr Soc* 2003, **51**(3):306-313.
22. Meyer G, Warnke A, Bender R, Muhlhauser I: **Effect on hip fractures of increased use of hip protectors in nursing homes: cluster randomised controlled trial**. *BMJ* 2003, **326**(7380):76.
23. O'Halloran PD, Cran GW, Beringer TR, Kernohan G, O'Neill C, Orr J, Dunlop L, Murray LJ: **A cluster randomised controlled trial to evaluate a policy of making hip protectors available to residents of nursing homes**. *Age Ageing* 2004, **33**(6):582-588.
24. Crotty M, Halbert J, Rowett D, Giles L, Birks R, Williams H, Whitehead C: **An outreach geriatric medication advisory service in residential aged care: a randomised controlled trial of case conferencing**. *Age Ageing* 2004, **33**(6):612-617.
25. Dyer CA, Taylor GJ, Reed M, Robertson DR, Harrington R: **Falls prevention in residential care homes: a randomised controlled trial**. *Age Ageing* 2004, **33**(6):596-602.
26. Crotty M, Whitehead C, Rowett D, Halbert J, Weller D, Finucane P, Esterman A: **An outreach intervention to implement evidence based practice in residential care: a randomized controlled trial [ISRCTN67855475]**. *BMC Health Serv Res* 2004, **4**(1):6.
27. Gold DT, Shipp KM, Pieper CF, Duncan PW, Martinez S, Lyles KW: **Group treatment improves trunk strength and psychological status in older women with vertebral fractures: results of a randomized, clinical trial**. *J Am Geriatr Soc* 2004, **52**(9):1471-1478.
28. Sloane PD, Hoeffer B, Mitchell CM, McKenzie DA, Barrick AL, Rader J, Stewart BJ, Talerico KA, Rasin JH, Zink RC *et al*: **Effect of person-centered showering and the towel bath on bathing-associated aggression, agitation, and discomfort in nursing home residents with dementia: a randomized, controlled trial**. *J Am Geriatr Soc* 2004, **52**(11):1795-1804.
29. Kerse N, Butler M, Robinson E, Todd M: **Fall prevention in residential care: a cluster, randomized, controlled trial**. *J Am Geriatr Soc* 2004, **52**(4):524-531.
30. Eisses AM, Kluiter H, Jongenelis K, Pot AM, Beekman AT, Ormel J: **Care staff training in detection of depression in residential homes for the elderly: randomised trial**. *Br J Psychiatry* 2005, **186**:404-409.
31. Loeb M, Brazil K, Lohfeld L, McGeer A, Simor A, Stevenson K, Zoutman D, Smith S, Liu X, Walter SD: **Effect of a multifaceted intervention on number of antimicrobial prescriptions for suspected urinary tract infections in residents of nursing homes: cluster randomised controlled trial**. *BMJ* 2005, **331**(7518):669.
32. Ray WA, Taylor JA, Brown AK, Gideon P, Hall K, Arbogast P, Meredith S: **Prevention of fall-related injuries in long-term care: a randomized controlled trial of staff education**. *Arch Intern Med* 2005, **165**(19):2293-2298.
33. Bravo G, Dubois MF, Roy PM: **Using goal attainment scaling to improve the quality of long-term care: a group-randomized trial**. *Int J Qual Health Care* 2005, **17**(6):511-519.

34. Testad I, Aasland AM, Aarsland D: **The effect of staff training on the use of restraint in dementia: a single-blind randomised controlled trial**. *Int J Geriatr Psychiatry* 2005, **20**(6):587-590.
35. Law M, Withers H, Morris J, Anderson F: **Vitamin D supplementation and the prevention of fractures and falls: results of a randomised trial in elderly people in residential accommodation**. *Age Ageing* 2006, **35**(5):482-486.
36. Hayward AC, Harling R, Wetten S, Johnson AM, Munro S, Smedley J, Murad S, Watson JM: **Effectiveness of an influenza vaccine programme for care home staff to prevent death, morbidity, and health service use among residents: cluster randomised controlled trial**. *BMJ* 2006, **333**(7581):1241.
37. Nijs KA, de Graaf C, Kok FJ, van Staveren WA: **Effect of family style mealtimes on quality of life, physical performance, and body weight of nursing home residents: cluster randomised controlled trial**. *BMJ* 2006, **332**(7551):1180-1184.
38. Rosendahl E, Lindelof N, Littbrand H, Yifter-Lindgren E, Lundin-Olsson L, Haglin L, Gustafson Y, Nyberg L: **High-intensity functional exercise program and protein-enriched energy supplement for older persons dependent in activities of daily living: a randomised controlled trial**. *Aust J Physiother* 2006, **52**(2):105-113.
39. Huizing AR, Hamers JP, Gulpers MJ, Berger MP: **Short-term effects of an educational intervention on physical restraint use: a cluster randomized trial**. *BMC Geriatr* 2006, **6**:17.
40. Loeb M, Carusone SC, Goeree R, Walter SD, Brazil K, Krueger P, Simor A, Moss L, Marrie T: **Effect of a clinical pathway to reduce hospitalizations in nursing home residents with pneumonia: a randomized controlled trial**. *JAMA* 2006, **295**(21):2503-2510.
41. Fossey J, Ballard C, Juszczak E, James I, Alder N, Jacoby R, Howard R: **Effect of enhanced psychosocial care on antipsychotic use in nursing home residents with severe dementia: cluster randomised trial**. *BMJ* 2006, **332**(7544):756-761.
42. Monette J, Miller MA, Monette M, Laurier C, Boivin JF, Sourial N, Le Cruguel JP, Vandal A, Cotton-Montpetit M: **Effect of an educational intervention on optimizing antibiotic prescribing in long-term care facilities**. *J Am Geriatr Soc* 2007, **55**(8):1231-1235.
43. Kovacs F, Abraira V, Santos S, Diaz E, Gestoso M, Muriel A, Gil del Real MT, Mufraggi N, Noguera J, Zamora J: **A comparison of two short education programs for improving low back pain-related disability in the elderly: a cluster randomized controlled trial**. *Spine (Phila Pa 1976)* 2007, **32**(10):1053-1059.
44. Kiel DP, Magaziner J, Zimmerman S, Ball L, Barton BA, Brown KM, Stone JP, Dewkett D, Birge SJ: **Efficacy of a hip protector to prevent hip fracture in nursing home residents: the HIP PRO randomized controlled trial**. *JAMA* 2007, **298**(4):413-422.
45. Sloane PD, Williams CS, Mitchell CM, Preisser JS, Wood W, Barrick AL, Hickman SE, Gill KS, Connell BR, Edinger J *et al*: **High-intensity environmental light in dementia: effect on sleep and activity**. *J Am Geriatr Soc* 2007, **55**(10):1524-1533.
46. Colon-Emeric CS, Lyles KW, House P, Levine DA, Schenck AP, Allison J, Gorospe J, Fermazin M, Oliver K, Curtis JR *et al*: **Randomized trial to improve fracture prevention in nursing home residents**. *Am J Med* 2007, **120**(10):886-892.

47. Orrell M, Hancock G, Hoe J, Woods B, Livingston G, Challis D: **A cluster randomised controlled trial to reduce the unmet needs of people with dementia living in residential care**. *Int J Geriatr Psychiatry* 2007, **22**(11):1127-1134.
48. Simmons SF, Keeler E, Zhuo X, Hickey KA, Sato HW, Schnelle JF: **Prevention of unintentional weight loss in nursing home residents: a controlled trial of feeding assistance**. *J Am Geriatr Soc* 2008, **56**(8):1466-1473.
49. Gurwitz JH, Field TS, Rochon P, Judge J, Harrold LR, Bell CM, Lee M, White K, LaPrino J, Erramuspe-Mainard J *et al*: **Effect of computerized provider order entry with clinical decision support on adverse drug events in the long-term care setting**. *J Am Geriatr Soc* 2008, **56**(12):2225-2233.
50. Riemersma-van der Lek RF, Swaab DF, Twisk J, Hol EM, Hoogendijk WJ, Van Someren EJ: **Effect of bright light and melatonin on cognitive and noncognitive function in elderly residents of group care facilities: a randomized controlled trial**. *JAMA* 2008, **299**(22):2642-2655.
51. Kerse N, Peri K, Robinson E, Wilkinson T, von Randow M, Kiata L, Parsons J, Latham N, Parsons M, Willingale J *et al*: **Does a functional activity programme improve function, quality of life, and falls for residents in long term care? Cluster randomised controlled trial**. *BMJ* 2008, **337**:a1445.
52. Bouwen A, De Lepeleire J, Buntinx F: **Rate of accidental falls in institutionalised older people with and without cognitive impairment halved as a result of a staff-oriented intervention**. *Age Ageing* 2008, **37**(3):306-310.
53. Peri K, Kerse N, Robinson E, Parsons M, Parsons J, Latham N: **Does functionally based activity make a difference to health status and mobility? A randomised controlled trial in residential care facilities (The Promoting Independent Living Study; PILS)**. *Age Ageing* 2008, **37**(1):57-63.
54. Bentzen H, Forsen L, Becker C, Bergland A: **Uptake and adherence with soft- and hard-shelled hip protectors in Norwegian nursing homes: a cluster randomised trial**. *Osteoporos Int* 2008, **19**(1):101-111.
55. Cox H, Puffer S, Morton V, Cooper C, Hodson J, Masud T, Oliver D, Preedy D, Selby P, Stone M *et al*: **Educating nursing home staff on fracture prevention: a cluster randomised trial**. *Age Ageing* 2008, **37**(2):167-172.
56. Gopal Rao G, Jeanes A, Russell H, Wilson D, Atere-Roberts E, O'Sullivan D, Donaldson N: **Effectiveness of short-term, enhanced, infection control support in improving compliance with infection control guidelines and practice in nursing homes: a cluster randomized trial**. *Epidemiol Infect* 2009, **137**(10):1465-1471.
57. Field TS, Rochon P, Lee M, Gavendo L, Baril JL, Gurwitz JH: **Computerized clinical decision support during medication ordering for long-term care residents with renal insufficiency**. *J Am Med Inform Assoc* 2009, **16**(4):480-485.
58. Huizing AR, Hamers JP, Gulpers MJ, Berger MP: **A cluster-randomized trial of an educational intervention to reduce the use of physical restraints with psychogeriatric nursing home residents**. *J Am Geriatr Soc* 2009, **57**(7):1139-1148.
59. Kuske B, Luck T, Hanns S, Matschinger H, Angermeyer MC, Behrens J, Riedel-Heller SG: **Training in dementia care: a cluster-randomized controlled trial of a training program for nursing home staff in Germany**. *Int Psychogeriatr* 2009, **21**(2):295-308.

60. Lemaitre M, Meret T, Rothan-Tondeur M, Belmin J, Lejonc JL, Luquel L, Piette F, Salom M, Verny M, Vetel JM *et al*: **Effect of influenza vaccination of nursing home staff on mortality of residents: a cluster-randomized trial**. *J Am Geriatr Soc* 2009, **57**(9):1580-1586.
61. Resnick B, Gruber-Baldini AL, Zimmerman S, Galik E, Pretzer-Aboff I, Russ K, Hebel JR: **Nursing home resident outcomes from the Res-Care intervention**. *J Am Geriatr Soc* 2009, **57**(7):1156-1165.
62. Meyer G, Kopke S, Haastert B, Muhlhauser I: **Comparison of a fall risk assessment tool with nurses' judgement alone: a cluster-randomised controlled trial**. *Age Ageing* 2009, **38**(4):417-423.
63. Patterson SM, Hughes CM, Crealey G, Cardwell C, Lapane KL: **An evaluation of an adapted U.S. model of pharmaceutical care to improve psychoactive prescribing for nursing home residents in northern ireland (fleetwood northern ireland study)**. *J Am Geriatr Soc* 2010, **58**(1):44-53.
64. Sackley CM, van den Berg ME, Lett K, Patel S, Hollands K, Wright CC, Hoppitt TJ: **Effects of a physiotherapy and occupational therapy intervention on mobility and activity in care home residents: a cluster randomised controlled trial**. *BMJ* 2009, **339**:b3123.
65. Eggermont LH, Knol DL, Hol EM, Swaab DF, Scherder EJ: **Hand motor activity, cognition, mood, and the rest-activity rhythm in dementia: a clustered RCT**. *Behav Brain Res* 2009, **196**(2):271-278.
66. Neyens JC, Dijcks BP, Twisk J, Schols JM, van Haastregt JC, van den Heuvel WJ, de Witte LP: **A multifactorial intervention for the prevention of falls in psychogeriatric nursing home patients, a randomised controlled trial (RCT)**. *Age Ageing* 2009, **38**(2):194-199.
67. Marcantonio ER, Bergmann MA, Kiely DK, Orav EJ, Jones RN: **Randomized trial of a delirium abatement program for postacute skilled nursing facilities**. *J Am Geriatr Soc* 2010, **58**(6):1019-1026.
68. Testad I, Ballard C, Bronnick K, Aarsland D: **The effect of staff training on agitation and use of restraint in nursing home residents with dementia: a single-blind, randomized controlled trial**. *J Clin Psychiatry* 2010, **71**(1):80-86.
69. Pellfolk TJ, Gustafson Y, Bucht G, Karlsson S: **Effects of a restraint minimization program on staff knowledge, attitudes, and practice: a cluster randomized trial**. *J Am Geriatr Soc* 2010, **58**(1):62-69.
70. Ward JA, Harden M, Gibson RE, Byles JE: **A cluster randomised controlled trial to prevent injury due to falls in a residential aged care population**. *Med J Aust* 2010, **192**(6):319-322.
71. Baldwin NS, Gilpin DF, Tunney MM, Kearney MP, Crymble L, Cardwell C, Hughes CM: **Cluster randomised controlled trial of an infection control education and training intervention programme focusing on meticillin-resistant Staphylococcus aureus in nursing homes for older people**. *J Hosp Infect* 2010, **76**(1):36-41.
72. van Gaal BG, Schoonhoven L, Vloet LC, Mintjes JA, Borm GF, Koopmans RT, van Achterberg T: **The effect of the SAFE or SORRY? programme on patient safety knowledge of nurses in hospitals and nursing homes: A cluster randomised trial**. *Int J Nurs Stud* 2010.
73. Looijmans-van den Akker I, van Delden JJ, Verheij TJ, van der Sande MA, van Essen GA, Riphagen-Dalhuisen J, Hulscher ME, Hak E: **Effects of a multi-faceted program to increase influenza vaccine uptake among health care workers in nursing homes: A cluster randomised controlled trial**. *Vaccine* 2010, **28**(31):5086-5092.

74. Jensen J, Nyberg L, Gustafson Y, Lundin-Olsson L: **Fall and injury prevention in residential care--effects in residents with higher and lower levels of cognition**. *J Am Geriatr Soc* 2003, **51**(5):627-635.

75. Warnke A, Meyer G, Bott U, Muhlhauser I: **Validation of a quality of life questionnaire measuring the subjective fear of falling in nursing home residents**. *Z Gerontol Geriatr* 2004, **37**(6):459-466.

76. Warnke A, Meyer G, Bender R, Muhlhauser I: **Predictors of adherence to the use of hip protectors in nursing home residents**. *J Am Geriatr Soc* 2004, **52**(3):340-345.

77. Jensen J, Nyberg L, Rosendahl E, Gustafson Y, Lundin-Olsson L: **Effects of a fall prevention program including exercise on mobility and falls in frail older people living in residential care facilities**. *Aging Clin Exp Res* 2004, **16**(4):283-292.

78. Sattin RW, Easley KA, Wolf SL, Chen Y, Kutner MH: **Reduction in fear of falling through intense tai chi exercise training in older, transitionally frail adults**. *J Am Geriatr Soc* 2005, **53**(7):1168-1178.

79. Bentzen H, Bergland A, Forsen L: **Risk of hip fractures in soft protected, hard protected, and unprotected falls**. *Inj Prev* 2008, **14**(5):306-310.

80. Rapp K, Lamb SE, Buchele G, Lall R, Lindemann U, Becker C: **Prevention of falls in nursing homes: subgroup analyses of a randomized fall prevention trial**. *J Am Geriatr Soc* 2008, **56**(6):1092-1097.

81. Rosendahl E, Gustafson Y, Nordin E, Lundin-Olsson L, Nyberg L: **A randomized controlled trial of fall prevention by a high-intensity functional exercise program for older people living in residential care facilities**. *Aging Clin Exp Res* 2008, **20**(1):67-75.

82. Huizing AR, Hamers JP, Gulpers MJ, Berger MP: **Preventing the use of physical restraints on residents newly admitted to psycho-geriatric nursing home wards: a cluster-randomized trial**. *Int J Nurs Stud* 2009, **46**(4):459-469.

83. Littbrand H, Lundin-Olsson L, Gustafson Y, Rosendahl E: **The effect of a high-intensity functional exercise program on activities of daily living: a randomized controlled trial in residential care facilities**. *J Am Geriatr Soc* 2009, **57**(10):1741-1749.

84. Conradsson M, Littbrand H, Lindelof N, Gustafson Y, Rosendahl E: **Effects of a high-intensity functional exercise programme on depressive symptoms and psychological well-being among older people living in residential care facilities: A cluster-randomized controlled trial**. *Aging Ment Health* 2010, **14**(5):565-576.
